# Advances in imaging techniques to assess kidney fibrosis

**DOI:** 10.1080/0886022X.2023.2171887

**Published:** 2023-02-01

**Authors:** Buchun Jiang, Fei Liu, Haidong Fu, Jianhua Mao

**Affiliations:** Department of Nephrology, The Children’s Hospital, Zhejiang University School of Medicine, National Clinical Research Center for Child Health, National Children’s Regional Medical Center, Hangzhou, China

**Keywords:** Imaging techniques, kidney fibrosis, ultrasound elastography, magnetic resonance imaging

## Abstract

As a sign of chronic kidney disease (CKD) progression, renal fibrosis is an irreversible and alarming pathological change. The accurate diagnosis of renal fibrosis depends on the widely used renal biopsy, but this diagnostic modality is invasive and can easily lead to sampling error. With the development of imaging techniques, an increasing number of noninvasive imaging techniques, such as multipara meter magnetic resonance imaging (MRI) and ultrasound elastography, have gained attention in assessing kidney fibrosis. Depending on their ability to detect changes in tissue stiffness and diffusion of water molecules, ultrasound elastography and some MRI techniques can indirectly assess the degree of fibrosis. The worsening of renal tissue oxygenation and perfusion measured by blood oxygenation level-dependent MRI and arterial spin labeling MRI separately is also an indirect reflection of renal fibrosis. Objective and quantitative indices of fibrosis may be available in the future by using novel techniques, such as photoacoustic imaging and fluorescence microscopy. However, these imaging techniques are susceptible to interference or may not be convenient. Due to the lack of sufficient specificity and sensitivity, these imaging techniques are neither widely accepted nor proposed by clinicians. These obstructions must be overcome by conducting technology research and more prospective studies. In this review, we emphasize the recent advancement of these noninvasive imaging techniques and provide clinicians a continuously updated perspective on the assessment of kidney fibrosis.

## Introduction

**Figure 1.**
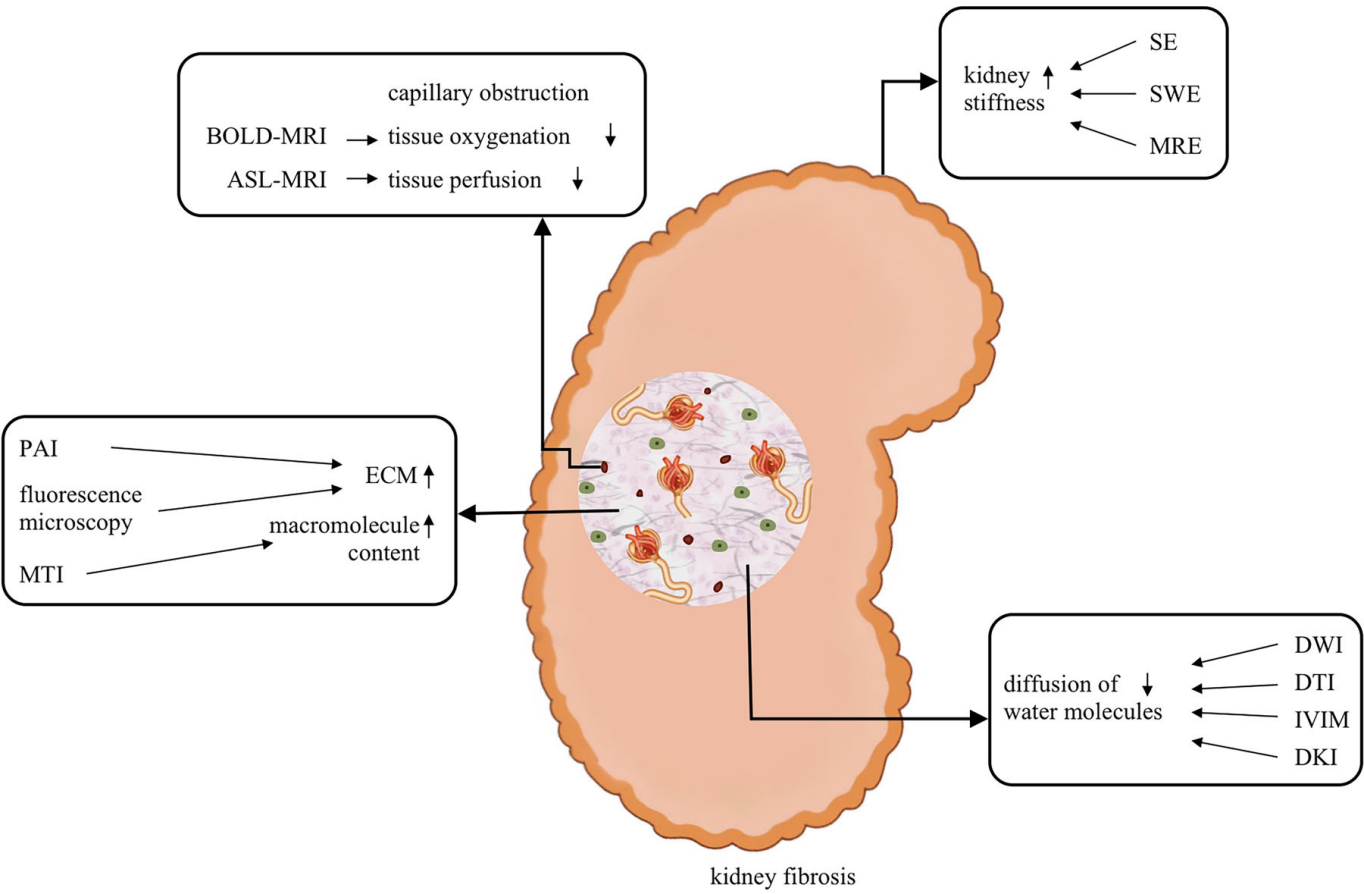
The pathological foundation of imaging techniques to assess kidney fibrosis. BOLD-MRI: Blood oxygenation level-dependent MRI; ASL-MRI: Arterial spin labeling MRI; SE: strain elastography; SWE: shear wave elastography; MRE: magnetic resonance elastography; PAI: photoacoustic imaging; ECM: extracellular matrix; MTI: magnetization transfer imaging; DWI: diffusion weighted imaging; DTI: diffusion tensor imaging; IVIM: intravoxel incoherent motion imaging; DKI: diffusion kurtosis imaging. The increase and deposition of ECM lead to the increase of kidney stiffness and macromolecule content, meanwhile, decrease the tissue oxygenation and perfusion, restrict the diffusion of water molecules. This pathologic change is the foundation of different imaging techniques to assess kidney fibrosis.

For patients with chronic kidney disease (CKD), renal fibrosis is of great concern because of its irreversible pathological process and it impairs renal function. Biopsy is the golden standard for diagnosing kidney fibrosis, nevertheless, it has limitation due to its invasiveness and sampling error. Hence, finding the best noninvasive imaging techniques to assess kidney fibrosis is important. Kidney fibrosis is characterized by extracellular matrix (ECM) proliferation, which is mainly produced by the increasing myofibroblasts [1,2]. During fibrosis, the size, structure, and composition of the kidney change, which are the research focuses and fundamental of imaging techniques. With the development of ultrasonography, magnetic resonance imaging (MRI), and fluorescence microscopy, these noninvasive imaging techniques have excellent application prospects in assessing kidney fibrosis. In this review, we summarize the features and studies of noninvasive imaging techniques (Figure 1, Tables 1 and 2) and discuss the clinical application of these techniques in kidney fibrosis.

**Table 1.** Features of noninvasive imaging techniques for assessing kidney fibrosis.

Method	Detection	index	Advantage	Disadvantage
SE	Tissue stiffness	Tissue mean elasticity	Simple to operate, cost effective	Not applied to assess native kidney
SWE	Tissue stiffness	SWV, Young’s modulus	Simple to operate, cost effective, apply to allografts and native kidneys	Influenced by tubular or interstitial pressure, tissue anisotropy and tissue perfusion
MRE	Tissue stiffness	renal shear stiffness	Faster acquisition sequence	Influenced by tissue perfusion, edema
DWI	Diffusion of water molecules	ADC	Simple to operate, get results quickly and easily	Influenced by the microcirculation perfusion and the blood flow of peritubular capillary
DTI	Diffusion of water molecules	FA	More sensitive and stable than ADC, can reflect the renal microstructural change	The detection process is interference by motion, such as respiratory movement
IVIM	Diffusion of water molecules	D, D*, f	Can separate the true water molecular diffusion	The detection process is more complicated and require higher signal-to-noise ratio
DKI	Diffusion of water molecules	K, D	Can reflect more complicated microstructural change	Lack of applications experiences in kidney fibrosis
BOLD-MRI	Tissue oxygenation	T2*, R2*	Can assess the tissue oxygenation noninvasively	Influenced by renal plasma flow and oxygen consumption. Cannot analysis the tissue hypoxia in detail
ASL-MRI	Tissue perfusion	cortical ASL perfusion; renal blood flow	Use the endogenous water of arterial blood as an endogenous tracer, can detect the cortical perfusion and renal blood flow	Lack of applications experiences in kidney fibrosis; cannot detect medullary blood flow; low signal-to-noise ratio and resolution
MTI	Macromolecule content	PSR, f	Can detect the macromolecule content in renal and quantify the degree of renal fibrosis to some extent; Few influenced by renal perfusion	Lack of applications experiences in human
PAI	The collagen content	the collagen content	Can quantify the collagen of kidney；can detect small vessels without contrast agent	Lack of applications experiences in human; insufficient measuring depth
Fluorescence microscopy	The collagen content	SHG	Can quantify the collagen of kidney；visual and dynamic observation of kidney fibrosis；highly sensitive, accurate and fast	Lack of applications experiences in human; need expensive and delicate instrumentations

SE: strain elastography; SWE: shear wave elastography; SWV: shear wave velocity; MRE: magnetic resonance elastography; DWI: diffusion weighted imaging; ADC: apparent diffusion coefficient; DTI: diffusion tensor imaging; FA: fractional anisotropy; IVIM: intravoxel incoherent motion imaging; D: true diffusivity; D*: pseudo diffusion coefficient; f: perfusion fraction; DKI: diffusion kurtosis imaging; K: the apparent diffusional kurtosis; D: the diffusion coefficient; BOLD-MRI: Blood oxygenation level-dependent MRI; T2*: the transverse relaxation time; R2*: the reciprocal of T2*; ASL-MRI: Arterial spin labeling MRI; MTI: magnetization transfer imaging; f: the bound pool fraction; PAI: photoacoustic imaging; SHG: second harmonic generation.

**Table 2.** Studies on noninvasive imaging techniques for assessing kidney fibrosis.

	Animal experiment	CKD	Renal allograft
SE	None	None	Tissue mean elasticity calculated from SE was negatively correlated with the degree of renal fibrosis [3]
SWE	SWE values had a positive correlation with kidney fibrosis in UUO model [4]	Kidney shear wave speed measured by ARFI was lower in patients with diabetic kidney disease or lower eGFR [5]; the mean Young’s modulus was found to be negatively correlated with eGFR [6]	kidney stiffness measured by TE was found significantly higher in renal allograft cortical interstitial fibrosis [7]
MRE	Shear stiffness measured by MRE increased with the progression of nephrocalcinosis in rat model [8]; renal medullary stiffness measured by MRE had a positive correlation with degree of fibrosis in pig model with renal artery stenosis [9]	renal stiffness values had a negative correlation with parameters of renal fibrosis in patients with glomerulonephritis and amyloid A amyloidosis [10]; the MRE values had a negative correlation with the interstitial extracellular matrix volume in patients with CKD [11];	Whole-kidney stiffness was positively associated with biopsy-derived fibrosis score and negatively associated with eGFR [12];
DWI	The ADC of kidney with an acute rejection was lower than kidney without rejection [13];	The renal ADC had a negative correlation with histological fibrosis score [14,15]	The ADC values of the patients with histologically proven evidence of acute rejection were lower than those with stable allograft function [16]
DTI	Cortex FA decreased in the early stage of renal fibrosis in rat models with diabetic nephropathy [17];	FA in the renal cortex and medulla had a significant negative correlation with glomerulosclerosis and tubulointerstitial fibrosis [18]	FA could detect allograft dysfunction early and had a negative correlation with the amount of renal fibrosis in patients after kidney transplantation [19]
IVIM	D, D*, and f values of the renal cortex and medulla decreased after obstruction in UUO model [20]	The values of f had a significantly negative correlation with interstitial fibrosis and tubular atrophy [21]	The values of f had a negative correlation with the time to recovery with respect to MRI and may predict the time to recovery in delayed graft function [22]
DKI	The mean D of cortex had a negative correlation with urea and alpha-SMA [23]	the renal parenchymal mean D values had a negative correlation with the histopathological fibrosis score, and the renal parenchymal mean K values had a positive correlation with histopathological fibrosis score [24];	None
BOLD-MRI	The T2* values were negatively correlated with the percentage of renal fibrotic area in a rabbit UUO model [25];	T2* was negatively correlated with fibrotic area [26]; the correlation between R2* and renal function was controversial	The cortical R2* values were positively associated with interstitial fibrosis in patients with allograft injury [27];
ASL-MRI	Mean cortical perfusion was higher in rats with chronic rejections than acute rejections, and the cortical renal blood flow was positively correlated with renal creatinine clearance [28]	The ASL perfusion decreased along with the exacerbation of fibrosis [29];	The combination of cortical ASL perfusion could effectively distinguish the allografts with subclinical pathology [30];
MTI	The renal fibrotic region had a higher pool size ratio [31,32]	None	None
PAI	2D PA imaging–based scores had a significant positive correlation with histological results [33]	None	None
Fluorescence microscopy	The SHG imaging found more collagen in the fibrotic kidney [34]; This HistoIndex platform could detect the collagen deposition of fibrotic kidney sensitively [35]	None	None

SE: strain elastography; SWE: shear wave elastography; ARFI: acoustic radiation force impulse; eGFR: estimated glomerular filtration rate; TE: transient elastography; MRE: magnetic resonance elastography; CKD: chronic kidney disease; DWI: diffusion weighted imaging; ADC: apparent diffusion coefficient; DTI: diffusion tensor imaging; FA: fractional anisotropy; IVIM: intravoxel incoherent motion imaging; DKI: diffusion kurtosis imaging; K: the apparent diffusional kurtosis; D: the diffusion coefficient; alpha-SMA: alpha-smooth muscle actin; BOLD-MRI: Blood oxygenation level-dependent MRI; T2*: the transverse relaxation time; R2*: the reciprocal of T2*; UUO: unilateral ureteral obstruction; R2*: the reciprocal of T2*; ASL-MRI: Arterial spin labeling MRI; MTI: magnetization transfer imaging; PAI: photoacoustic imaging; SHG: second harmonic generation.

### Ultrasonography

Along with an increase in ECM during fibrosis, the elasticity of renal tissue changes, which is the detecting principle of ultrasonography [36]. Traditional ultrasonography is mainly used for detecting kidney stones and renal space-occupying diseases; however, it provides little help for detecting renal fibrosis. With the development of ultrasonography, ultrasound elastography offers a new perspective on renal fibrosis. Two kinds of ultrasound elastography, namely, strain elastography (SE) and shear wave elastography (SWE), are mainly used.

### Strain elastography

The principle of SE is that applying pressure to the kidney can make a displacement, and then, a transducer can obtain images with colors. The different colors in the images represent different stiffness [37]. The completion of SE requires external pressure compression; therefore, it does not apply to the kidneys located at deeper areas of the abdomen. Studies on SE in renal fibrosis focused on renal allografts, which are closer to the body surface [3,36,38]. A small study showed that the corticomedullary strain ratio from SE is a better method for assessing renal allograft cortical interstitial fibrosis/tubular atrophy than Doppler parameters [38]. Two other studies conducted by the same research team showed that the corticomedullary strain ratio and normalized cortical strain on SE may be a good noninvasive method for detecting renal fibrosis [39,40]. A prospective study on graft fibrosis found that the tissue mean elasticity calculated from SE was negatively correlated with the degree of renal fibrosis, and the accuracy of diagnosing moderate to severe fibrosis could reach as high as 95% [3]. Hence, SE is a promising method for assessing renal allograft cortical interstitial fibrosis.

### Shear wave elastography

SWE is another ultrasound elastography technique, which can detect tissue stiffness using ultrasound-generated shear wave velocity (SWV), similar to a virtual ‘finger’. Tissue stiffness is directly proportional to the square of the SWV. SWE can assess kidney stiffness without external pressure compression and can be applied to native kidneys, not just renal allografts [41,42]. An experiment involving animal models of unilateral ureteral obstruction (UUO) found that SWE values had a positive correlation with kidney fibrosis, which was quantified by Picrosirius red and Masson trichrome [4]. Therefore, an increasing number of researchers have focused on the application value of SWE in patients with renal fibrosis. A prospective study found that Young ’s modulus measured by SWV, an index reflecting the tissue stiffness, had high specificity and sensitivity in the diagnosis of interstitial fibrosis in patients with IgA nephropathy [43].

There are three types of this imaging technique to be used in kidney diseases: acoustic radiation force impulse (ARFI), supersonic shear imaging, and transient elastography. ARFI was found to be a possibly effective and promising method for assessing CKD and renal fibrosis [44,45]. A study found that ARFI may be a method for predicting acute rejection in patients undergoing kidney transplantation [46]; however, this study lacked adequate evidence. A prospective study showed that SWV measured using ARFI was lower in patients with diabetic nephropathy or lower estimated glomerular filtration rate (eGFR) but was unrelated to proteinuria level [5]. Aimed to compare the renal cortical stiffness between individuals with CKD and healthy people, supersonic shear imaging was performed in 32 patients with CKD and 20 healthy individuals. Young’s modulus, the index that increases with tissue stiffness, was higher in the CKD group [47]. In another study, the mean Young ’s modulus was negatively correlated with eGFR, and supersonic shear imaging was expected to be a potential index for diagnosing and staging diabetic kidney disease [6]. Because of the deep location of native kidneys, studies of transient elastography focused on kidney allografts [7,48,49], similar to studies on SE. Kidney stiffness measured by using transient elastography was significantly higher in renal allograft cortical interstitial fibrosis [7].

However, there are different views on the effect of ultrasound elastography in renal fibrosis. Some studies found no significant correlation between SWV and renal fibrosis and CKD stage [50–53]. Renal stiffness is influenced by several factors, not only fibrosis. First, age, body mass index, gender, and individual variations [54] can influence renal stiffness, which was reported in some studies [7,47,53–55]. Second, renal perfusion is a crucial factor. Animal experiments showed that renal artery and vein ligation can influence renal elasticity values [56]. Aiming to explore the influence of renal perfusion on SWV values, a study measured the SWV and brachial–ankle pulse wave velocity simultaneously in 183 patients with CKD. The brachial–ankle pulse wave velocity, which represents arteriosclerosis of large vessels, was negatively correlated with SWV. In other words, patients with arteriosclerosis of large vessels, which decreases renal perfusion, may have a lower SWV [55]. Additionally, the inhomogeneity of renal fibrosis, the difference in kidney perfusion between the cortex and medulla, and the cooperation degree of patients are also factors that must be considered [57]. SWE is a promising technique for evaluating kidney fibrosis; it is noninvasive and easy to operate but needs more studies to confirm the correlation between renal stiffness indexes and fibrosis.

### Photoacoustic imaging

Photoacoustic imaging (PAI) is a new noninvasive technique that combines ultrasound and laser without radiation. PAI can use ultrasound to detect the acoustic signals produced by the thermoelastic expansion of tissues, which is caused by laser irradiation. Because of the different light-absorption spectra of tissue components, PAI can quantify the collagen content in the kidneys and reflect the progression of fibrosis.

Animal studies confirmed that PAI could detect the blood oxygen saturation in patients with acute kidney injury [58] and the kidney vasculature in patients with polycystic kidney disease [59]. Recently, a study on PAI of collagen in animals and humans showed that two-dimensional PAI-based scores had a significant positive correlation with histological results, and alpha-smooth muscle actin was considered a marker of kidney fibrosis in mice with UUO. Moreover, a similar result was found in human radical nephrectomy specimens [33]. This study displayed the enormous potential of PAI in quantifying collagen content and assessing renal fibrosis. Nevertheless, the lack of clinical evidence and the increasing detection depth are big problems that must be solved.

## MRI

### Diffusion MRI

The motion of water molecules in the body is restricted by fibers, macromolecules, and other tissue components. Renal fibrosis can influence the water diffusion pattern in the kidney, which is the application principle of diffusion MRI in kidney diseases. Generally, the following are common manners of diffusion MRI: diffusion-weighted imaging (DWI), diffusion tensor imaging (DTI), intravoxel incoherent motion imaging (IVIM), and diffusion kurtosis imaging (DKI).

### Diffusion-weighted imaging

The main detection index of DWI used in renal fibrosis is the apparent diffusion coefficient (ADC), which indicates the water motion in renal tissue. Fibrosis can cause vascular structure destruction and extracellular matrix proliferation, which influence the blood flow perfusion and water motion, which decreases the ADC. An animal experiment showed that in renal allografts, the ADC of the kidneys with an acute rejection was lower than those without rejection [13]. Furthermore, a similar result was found in patients after kidney transplantation [16,60]. Besides renal allografts, several studies have demonstrated that ADC is also effective in assessing fibrosis of native kidneys [14,15,26,61–63]. A latest prospective study of DWI involving patients with CKD or kidney allograft, found that the cortico-medullary difference of ADC is a more excellent predictor of interstitial fibrosis and kidney function decline than ADC in the cortex or medulla alone [64].

Although the negative correlation between ADC and kidney fibrosis was confirmed by many studies, some defects must be considered. Similar to the SWV of ultrasound elastography, ADC is also influenced by the microcirculation perfusion and the blood flow of peritubular capillary, not only fibrosis. Animal experiments found that the ADC in the cortex and medulla decreased along with the reduction of renal perfusion during the injection of angiotensin II [65]. A similar result was observed in other animal experiments and patients with renal artery stenosis [66,67]. An experiment involving animal models of UUO found that the ADC in rats with renal fibrosis was significantly increased at postmortem and higher than in healthy kidney parenchyma, which was because of tubular dilation and interstitial expansion [68]. Hence, the decrease in ADC may be an indicator of low renal perfusion or worsening of renal function caused by fibrosis, not fibrosis itself. The variations in the results demonstrate the imperfection of ADC and a better indicator which will be unaffected by renal perfusion is needed.

### Diffusion tensor imaging

DTI is a new diffusion MRI that can detect the anisotropy of water molecular diffusion based on DWI and paint a picture of changes in the renal microstructure. The main index of DTI is fractional anisotropy (FA). Renal fibrosis can lead to interstitial fibrosis, glomerulosclerosis, and inflammatory infiltration, which cause FA changes [69–71]. An animal experiment on diabetic nephropathy showed that the cortex FA decreased in the early stage of renal fibrosis. Interestingly, the ADC values of rat models with diabetic nephropathy increased after 12 weeks, in contrast to FA. After 24 weeks, both decreased [17]. This might be because the effect of kidney hypertrophy and hyperfiltration was stronger than that of mild fibrosis. Studies on DTI of native kidney or renal allografts found that FA was negatively correlated with the amount of renal fibrosis [18,19,72–74]. Additionally, a study showed that the medulla FA is higher than the cortex FA, contrary to ADC [75,76]. And FA may be influenced by the transport of water molecules in the collecting tubules [76]. FA seems to be more sensitive and stable than ADC [17,77].

### Intravoxel incoherent motion imaging

IVIM, a supplement and optimization for ADC, can separate out the pure molecule diffusion and assess kidney capillary perfusion [78]. The main parameters of IVIM include true diffusivity (D), pseudo diffusion coefficient (D*), and perfusion fraction (f). The value of D is a parameter reflecting intracellular and intercellular water molecular movement, and D* can assess microcirculation in vessels or perfusion [20,79]. Meanwhile, the value of f indicates the total tissue perfusion, including all blood flows to capillaries [80]. Moreover, IVIM can separate the true water molecular diffusion by the biexponential fitting of DWI data and detail the mechanism of diffusion without the influence of tissue perfusion [81].

Experiments on animal renal fibrosis models showed correlations between the reduction of IVIM parameters and renal fibrosis [20,22,25,82]. A prospective study on IVIM in chronic kidney disease, aimed to explore its contributions to assess renal function and pathological changes, showed that the values of D and D* were negatively correlated with the stage of CKD and the values of f. The values of D and f had a positive correlation with eGFR. Additionally, it was suggested that the values of f had a significantly negative correlation with the total renal pathological score, which included interstitial fibrosis and tubular atrophy [21]. The same results were observed in other clinical studies of renal fibrosis [83,84]. Therefore, both animal and clinical studies showed the tremendous potential and superiority of IVIM in assessing kidney fibrosis.

### Diffusion kurtosis imaging

Fibrosis can change the cell membranes and produce more ECM, which can obstruct the free diffusion of water molecules, resulting in a non-free movement, called non-Gaussian distribution. Non-Gaussian distribution is precisely what DKI aims to detect [85,86]. There are two parameters of DKI: apparent diffusional kurtosis (K), which reflects the peak distribution of tissue diffusivity, and diffusion coefficient (D) under a non-Gaussian distribution similar to ADC [86]. Higher values of K and lower values of D indicate a greater influence on the movement of water molecules, which may reflect the greater damage to the renal parenchyma.

DKI is a novel technique, and only a few studies have evaluated its value in assessing renal fibrosis. Early animal models of renal fibrosis and studies of healthy volunteers confirmed that DKI is feasible in human kidneys [85,87] and can be used to assess renal fibrosis in animal models [23]. Recent studies on DKI in patients with CKD showed a negative correlation between the mean D values in the renal parenchyma and the histopathological fibrosis score, whereas the mean K values in the renal parenchyma showed a positive correlation with the histopathological fibrosis score [24]. The same group also conducted a prospective study to compare the diagnostic efficacy between DKI and DWI in renal fibrosis. In that study, the mean K values in the renal parenchyma were positively associated with the total pathological renal injury score, which included interstitial fibrosis and tubular atrophy. Additionally, the diagnostic efficacy of K was found to be superior to that of ADC in distinguishing between mild renal injury and moderate–severe renal injury [88]. Studies of DKI in renal tissue are still scarce, and the current experiences demonstrate that this new technique deserves more researches and concerns.

### Blood oxygenation level-dependent MRI

In the progression of renal fibrosis, the decrease in peritubular capillary and oxygen quantity is an important characteristic [89,90]. Due to the different magnetism of oxygenated and deoxygenated hemoglobin, hypoxia can influence the proportions of oxygenated and deoxygenated hemoglobin, resulting in changes in the magnetic field characteristic. This pathological change is just the fundamental of Blood oxygenation level-dependent MRI (BOLD-MRI). The main parameter of BOLD-MRI to assess tissue oxygenation is the transverse relaxation time (T2*) or the reciprocal of T2* (R2*).

According to the aforementioned pathological change, Ogawa et al. first reported BOLD-MRI as a new technique for assessing tissue oxygenation [91]. Subsequently, Prasad et al. first used BOLD-MRI to assess renal tissue oxygenation and found the feasibility of this technique in the human kidney [92]. A study of animal UUO models showed that the T2* values were negatively correlated with the percentage of renal fibrotic area [25]. Similar results were found in other animal experiments [93–95]. Additionally, a study of animal UUO models found that the R2* values did not increase and were kept steady after 6 weeks, indicating that BOLD-MRI has little effect on the long-term assessment of renal fibrosis [94].

Thus far, studies on BOLD-MRI in kidney disease focused on the predictive effect and monitoring of CKD; however, the results are controversial. A controlled study found that the R2* values are higher in patients with CKD than in healthy individuals and are positively correlated with serum creatinine and blood urea nitrogen. Additionally, a study showed that the R2* values were negatively correlated with eGFR and effective renal plasma flow [96]. Comparable results have been found in other studies of CKD [97,98]. However, a few studies found that the parameter of BOLD-MRI had no relationship to kidney function [99,100] or eGFR [100–102].

The application of BOLD-MRI in patients with renal fibrosis is limited but has more satisfying results than in patients with CKD. Inoue et al. used DWI and BOLD-MRI to assess renal fibrosis and hypoxia of the cortex in patients with diabetic nephropathy, non-diabetic CKD, and acute kidney injury. Consistent with the results of animal UUO models, it was suggested that the values of ADC and T2* were negatively correlated with the fibrotic area [26]. A recent study on BOLD-MRI in kidney allografts found that the cortical R2* values were positively associated with interstitial fibrosis in patients with allograft injury [27]. The different results of BOLD-MRI in CKD suggest that the R2* or T2*values are susceptible. The effective renal plasma flow and oxygen consumption caused by different renal diseases may influence the accuracy of BOLD-MRI. As studies related to renal fibrosis are limited, the potential of BOLD-MRI in assessing renal fibrosis remains to be elucidated.

### Arterial spin labeling MRI

Arterial spin labeling (ASL) MRI uses the endogenous water of arterial blood as an endogenous tracer to detect tissue perfusion. Because of the affluent blood supply of the renal cortex, the cortical ASL perfusion or renal blood flow is the main indicator of this technique. Earlier studies found that ASL perfusion could reflect and quantify the perfusion in native and transplanted kidneys [103,104]. An animal experiment of ASL-MRI in rats after renal transplantation showed that the mean cortical perfusion was higher in rats with chronic rejections than in those with acute rejections, and the cortical renal blood flow was positively correlated with renal creatinine clearance [28]. Subsequently, several studies confirmed that ASL perfusion had a significantly positive correlation with eGFR in CKD, including diabetic nephropathy [105–107], and in transplanted kidneys [108].

Studies on the application of ASL-MRI in renal fibrosis are still limited. A pilot study, which combined magnetic resonance elastography (MRE) and ASL to assess fibrosis in diabetic nephropathy, indicated that ASL perfusion decreased along with the exacerbation of fibrosis [29]. A similar result was found in a study on renal allograft fibrosis [109]. A single-center prospective study, aimed to explore the effect of ASL-MRI in kidney allografts with stable graft function, found that the combination of the cortical ASL perfusion could effectively determine allografts with subclinical pathology, which included interstitial fibrosis, peritubular capillaritis, and tubular atrophy [30]. Because of the influence of low signal-to-noise ratio and resolution, as well as the limited experiences in kidney fibrosis, ASL-MRI has not been widely used in renal diseases, despite its enormous potential. Future studies on the determination method improvement are needed.

### Magnetic resonance elastography

MRE relies on the detection of shear wave diffusion caused by mechanical vibrations to reflect tissue stiffness. MRE has been widely used in liver fibrosis [110,111]; however, its use in kidney fibrosis has been limited. Studies confirmed the feasibility and reliability of MRE in assessing renal stiffness [112]. A study of rat models with nephrocalcinosis showed that the shear stiffness measured using MRE increased with the progression of nephrocalcinosis [8]. Another study involving pig models with renal artery stenosis indicated that renal medullary stiffness measured using MRE had a positive correlation with the degree of fibrosis [9]. Moreover, another animal study found that renal medullary stiffness is significantly associated with the degree of fibrosis [113].

Compared with those of animal studies, the results of renal fibrosis studies in humans are inconsistent. A prospective cohort study of MRE in kidney allografts found that whole-kidney stiffness was positively associated with biopsy-derived fibrosis score and negatively associated with eGFR [12], which were also observed in other studies of kidney allografts and native kidneys [114–116]. Conversely, a recent study on MRE in patients with glomerulonephritis and amyloid A amyloidosis demonstrated that renal stiffness values are negatively correlated with the parameters of renal fibrosis. Meanwhile, the worse the renal function, the lower the renal stiffness values [10]. A decrease in MRE shear stiffness has also been found in studies on diabetic nephropathy [29], IgA nephropathy [117], and lupus nephritis [118]. Additionally, a recent study of MRE in patients with CKD showed that the MRE values are negatively correlated with the interstitial extracellular matrix volume. Moreover, this study drew the best mapping model as follows: interstitial extracellular matrix volume = 218.504–14.651 × In (MRE)–18.499 × In(eGFR) [11]. The opposite result of MRE in renal fibrosis is explained by the influence of various factors, including renal perfusion, the deep location of the kidney, and the heterogeneity of the fibrotic kidney. Several studies confirmed that the MRE shear stiffness could be influenced by renal perfusion and had a positive correlation [119–121]. Hence, the aforementioned factors must be considered when MRE is used to assess renal fibrosis, particularly the renal perfusion pressure.

### Magnetization transfer imaging

Magnetization transfer imaging (MTI) is aimed principally at detecting increases in macromolecules in renal fibrosis. According to the different magnetization between free water molecules and water molecules combined with ECM, MTI can reflect the macromolecule content in the renal parenchyma, which indicates renal fibrosis indirectly. The index of MTI is the MT ratio (MTR), which can indicate the macromolecule content in the renal parenchyma. Several animal studies confirmed that MTR is positively associated with renal fibrosis [122–124]. Additionally, an animal experiment found that MTR was little influenced by renal perfusion, indicating the reliability of MTR in assessing renal fibrosis [125].

Quantitative MTI is a superior technique developed from the traditional MTI, which can more accurately quantify the macromolecule content of tissues. The main parameters of quantitative MTI are the pool size ratio, which is the ratio between the bound and free pool magnetization, and the bound pool fraction (f), which reflects the fraction of bound pool in tissues. A study involving murine models with kidney fibrosis found that the renal fibrotic region had a higher pool size ratio, particularly in the outer stripe of the outer medulla and cortex [31]. The cortical regions with a higher pool size ratio were significantly correlated with fibrosis in murine models of diabetic nephropathy [32]. Additionally, animal studies on renal artery stenosis found the great potential of the f values in assessing renal fibrosis, which were immune from the effects of tissue specificity, such as renal perfusion and elasticity [126,127]. Nevertheless, MTI has not been used in clinical practice, and studies involving patients with renal diseases are required.

### Other MRI techniques

Furthermore, some other MRI techniques contribute to the assessment of renal fibrosis, such as native T1 mapping, susceptibility-weighted imaging (SWI), and elastin-specific MRI.

Animal experiments of native T1 mapping showed that T1 relaxation times, which represent the spin-lattice, is positively correlated with the degree of renal fibrosis [63,128]. In patients with chronic glomerulonephritis, the T1 relaxation times were also positively associated with the glomerular, vascular, tubulointerstitial, and interstitial fibrosis scores [129]. However, no relationship between T1 relaxation times and histological results was found in patients with IgA nephropathy [130]. T1 relaxation times were not only influenced by fibrosis but also affected by edema and inflammation, this may limit its application.

The studies on SWI in renal fibrosis were mainly preclinical studies, such as UUO models [131,132]. It was found that the cortical and medullary r values from SWI were significantly inversely associated with the renal fibrosis scores [132]. A study on SWI with small sample sizes of type 2 diabetes mellitus suggested that the parameters of SWI were significantly correlated with renal function indexes [133]. Because of the limited studies, there is still a long way to go before the clinical application of SWI.

A recent study on elastin-specific MRI in UUO rat models and patients with various kidney diseases showed that the expression of elastin detected using elastin-specific MRI agent (ESMA) could monitor and quantify kidney fibrosis, and ESMA-based molecular MRI is a promising technique for assessing kidney fibrosis [134]. Nevertheless, there are some obstructions that must be overcome, such as the safety of contrast agents in end-stage renal disease and the lack of clinical evidence [135].

### Fluorescence microscopy

Fluorescence microscopy is one of the research focuses currently on *in vivo* imaging, particularly two-photon excitation microscopy. This new technique can detect second harmonic generation (SHG), which is produced by the interaction of light with collagen. Thus, kidney fibrosis can be quantified by detecting SHG, which reflects the content of collagen [136]. The deep imaging *via* emission recovery detector is a novel technique that helps form a label-free, deep, and highly sensitive imaging of SHG.

Recently, studies on fluorescence microscopy are limited to animal models. A study involving murine UUO models showed that SHG imaging found more collagen in the fibrotic kidney; the combination of SHG imaging and fluorescence lifetime imaging is a promising technique that can accurately assess the degree of kidney fibrosis [34]. Another study using HistoIndex’s Genesis200 platform in mice with UUO, which combines SHG imaging and two-photon excitation microscopy, showed that HistoIndex’s Genesis200 platform could sensitively detect the collagen deposition of the fibrotic kidney and is be a good supplement to traditional pathological examination [35]. Studies on fluorescence microscopy are still in the preclinical stage; however, they show the possibility of visual and dynamic observation of kidney fibrosis.

## Conclusions

As is known to all, biopsy is the golden standard for diagnosing kidney fibrosis. And the development of pathological microscopic image can also take the furthest advantage of renal biopsy. However, its invasiveness and sampling error are still considerable [137]. The development of noninvasive imaging techniques, such as MRI and ultrasonography, offers an increasing number of methods for detecting and monitoring kidney fibrosis. In patients with kidney fibrosis, changes in tissue stiffness and the water diffusion pattern can be detected using ultrasound elastography and DWI. As novel MRI techniques, ASL-MRI and BOLD-MRI can separately detect changes in tissue perfusion and oxygenation to indirectly reflect kidney fibrosis. Fluorescence microscopy and PAI are both promising techniques, which may quantify the degree of fibrosis; however, their application is still in the research phases. The aforementioned imaging techniques not only help in diagnosing and monitoring kidney fibrosis but also contribute to evaluating the prognosis and effects of this disease.

For nephrologists, the more techniques, the harder it is to choose. Future studies should subdivide the disease spectrum of kidney fibrosis and choose an individualized detection method. Based on the current experiences, different imaging techniques should be selected for different diseases, not one fits all. For example, ASL-MRI and BOLD-MRI, which aim to detect tissue perfusion and oxygenation, may have priority in patients with ischemic kidney diseases, such as renal artery stenoses. Because of their convenience and flexibility, SE and SWE may be more suitable for risk screening and follow-up of kidney fibrosis. Multiparameter MRI can offer a more comprehensive understanding of the risk and degree of kidney fibrosis. PAI and fluorescence microscopy, which can quantify the collagen content, may be more popular in animal studies, at least for now. For clinicians, an individualized detection method is a vision for the future, and further studies are needed to tell them how to choose these noninvasive techniques for all kinds of kidney diseases.

Furthermore, the immaturity of detection and quality control has produced some conflicting results, which have confused numerous clinicians. Some values of these novel techniques, such as ADC and index of BOLD-MRI, can be influenced by kidney perfusion. In the preparation for imaging, any factors which can influence the kidney perfusion should be considered, such as acute disease (diarrhea or shock) and drugs (angiotensin-converting enzyme inhibitor or angiotensin II receptor blocker). Aimed to ensure accuracy, the application of these techniques in clinical practice should avoid these disturbing factors. Strict operating standards and quality controls are required to ensure the accuracy and reproducibility of these techniques. In addition, the heterogeneity of the fibrotic kidney is also an important factor to influence the accuracy of these techniques. Due to the subjectivity and sampling error, renal biopsy in human studies is difficult to guarantee the accuracy. Whole-kidney fibrosis detection can only be performed in animal studies, which provide important basic data for human studies. Also, the increase of sample size may minimize this influence in human studies.

Except for PAI, MTI, and fluorescence microscopy, most imaging techniques mentioned above have been used in clinical practice and studies nowadays. Ultrasound elastography and DWI have broader applications than other imaging techniques, but are still limited in kidney fibrosis. With the development of these noninvasive imaging techniques for kidney fibrosis, deepening the knowledge of these novel assessment approaches is necessary for nephrologists.
